# Virtual reality, face-to-face, and 2D video conferencing differently impact fatigue, creativity, flow, and decision-making in workplace dynamics

**DOI:** 10.1038/s41598-024-60942-6

**Published:** 2024-05-04

**Authors:** Gregorio Macchi, Nicola De Pisapia

**Affiliations:** Department of Psychology and Cognitive Science (DIPSCO), University of Trento, Corso Bettini 31, 38068 Rovereto, TN Italy

**Keywords:** Human behaviour, Psychology

## Abstract

Digital communication technologies are rapidly evolving, and understanding their impact on group dynamics and cognitive performance in professional settings becomes central. This study investigates the psychological impact of different interaction settings—two-dimensional Video Conferencing (VC), Face-To-Face (FTF), and Virtual Reality (VR)—on group dynamics, cognitive performance, and aspects of well-being in a professional context. Utilizing a sample of 40 participants from a large Italian electricity transmission company, the study employs a within-subjects design to explore various metrics, including flow, creativity, fatigue and aspects of interaction. The results indicate that FTF interactions are optimal for idea generation and task absorption. VR, although initially more fatiguing for first-time users, fosters a more collaborative and peaceful environment, encouraging participants to engage more openly with each other. VC was found to be the least fatiguing, but also the least engaging in terms of task absorption and idea generation. Additionally, age-related differences were observed, particularly in the perception of motivational and emotional fatigue in the VR setting. The study provides empirical evidence supporting the integration of VR in professional settings for specific types of meetings, while also highlighting the limitations and areas for future research. These findings have implications for organizational well-being, cognitive ergonomics, and the evolving landscape of remote work technologies.

## Introduction

The advent of technology has revolutionized the way we work, providing options beyond the traditional physical office space. While two-dimensional remote working Video Conferencing (VC) platforms are increasingly common, advancements in immersive environments in Virtual Reality (VR) are opening new dimensions for work settings. Despite growing research on VC remote working^1^, literature comparing it with VR and Face-To-Face (FTF) settings is very limited, as we describe in the first part of this introduction. In the second part of the introduction, we illustrate the premises of how this study aims to fill this gap by examining decision-making processes under these three conditions.

In the last years, VR technology has seen a significant expansion in its use not only by researchers^2–4^, but also by organizations and private companies, for example for training^5^, thanks also to the increase in the accessibility of devices, in economic and diffusion terms. However, this type of technology has existed for many years in more rudimentary forms and its use is a source of interest for researchers in psychology and neuroscience, driven to learn about their potential and their influence on the user who uses them. As reported in Bohil et al.^2^, the goal of the current form of VR is to stimulate brain and behavioral responses in the virtual environment that are analogous to those experienced in the real world.

In VR, the user immerses himself/herself using hardware components that are usually composed of a specific viewer that allows stereoscopic views of the scene based on position and orientation, obtaining the sensation of his/her point of view. The headset isolates users from outside stimuli, further orienting the users’ focus on the task at hand and making them immersed in the virtual world and less prone to distraction from their surroundings. Immersive environments in VR induce in the users a sense of presence, which is the sensation of being physically present in the simulated space. This experience is achieved through a combination of audio, visual, and sometimes tactile elements, which together create a sense of being within an artificial 3D environment. Controllers are used to interact with the environment in which the user is immersed. This is in contrast to less immersive experiences, such as watching a film, where there's still an awareness of the surrounding physical world^6–8^.

A more recent fundamental aspect of VR worlds is to give the possibility to interact with other people while connected from different parts of the physical world. To do this, users must create their own online identity. This is achieved through a three-dimensional representation of themselves, or of an alternative self, i.e. an avatar^9^. This word comes from Sanskrit, that indicates the human manifestation of a deity on Earth^10^. The sensation of representing ourselves in an avatar is important to nourish the sense of psychological presence in what we do in the virtual environment, to support deep levels of collaboration, communication and relationship building^11,12^.

VR has found applications in diverse fields:^4^ it is used in games and video games^13,14^, in education^15^, for military exercises and training^16,17^, in architecture for the design of structures^18^, to train and transmit social skills^19^, in medicine to carry out simulations of the different innovative surgical procedures^20^, and to assist the elderly and for different treatments in the psychological field^21,22^. It is increasingly being adopted in corporate settings, but research on its optimal use in such environments is still in its infancy^5^.

This study, therefore, aims to contribute to this underexplored area by focusing on psychological factors that significantly impact organizational performance. We explore whether VR, VC and FTF can differentially and significantly influence mental constructs that are key in such contexts, namely decision-making, creativity, and flow. Focusing on these three constructs is vital in today's dynamic business landscape. Decision-making is critical for both strategic and operational choices; creativity is essential for organizational innovation and problem-solving; flow, the deep immersion in tasks, is key to enhancing workplace performance and satisfaction^23^ How different settings influence these constructs is crucial for optimizing workplace effectiveness and fostering an environment conducive to innovation.

Regarding related work on the psychological effects induced by digital environments, a previous comparison between VR and VC in decision making showed that accuracy and time did not differ significantly^24^, while in another study the participants spent a longer average time for the decision making task in VR^25^ (however, in this case, the researchers did not perform a statistical test for comparison). In the comparison of the three conditions (VR, VC and FTF), the quality of the discussion, the cognitive load and the probability of solving the task correctly did not differ between the VR and the other two. The social presence in VR was lower than that perceived FTF, but still evaluated with higher levels than in the VC mode^26^.

When it comes to examining creativity across different interaction settings, existing literature predominantly focuses on specialized domains such as graphic design or artistic endeavors^27^. Previous empirical evidence suggests that immersive VR offers a distinct advantage over traditional 2D platforms. Participants in VR settings report a heightened sense of playfulness and find the environment conducive to focused activity by minimizing distractions. VR has the potential to bring workers in environments that facilitate more uninhibited interactions and foster a relaxed atmosphere, thereby promoting responsive collaboration. However, it is crucial to note that VR is not without its limitations, such as the absence of some elements of interpersonal communication that are naturally present in FTF interactions^28^.

In a preliminary study conducted by Petrykowski et al.^25^ the VR setting yielded a greater number of idea-laden post-its compared to the video call environment. It is important to qualify this finding by noting that the researchers did not perform a statistical comparison to substantiate the observed differences. Ide et al.^29^ conducted a nuanced investigation into the role of non-verbal cues—specifically gestures and facial expressions—in collaborative interactions. The study employed a controlled experimental design featuring three distinct conditions: VR with the inclusion of symbolic gestures, VR without symbolic gestures, and traditional FTF interaction. A range of metrics was employed to evaluate the outcomes, including the quantity of ideas generated during brainstorming sessions, the duration of each interaction, and the balance and frequency of participant contributions, which were recorded via microphones. Additionally, the study quantified the utilization of symbolic gestures and collected questionnaire data for further insights. Intriguingly, their findings revealed no statistically significant differences across the three conditions in terms of idea generation, interaction duration, or the balance of participation among team members.

Our research attempts to fill a gap in the psychological literature by comparing the efficacy of VR, VC and in-person meetings, particularly in the context of creative group interactions. To inform our study design, we conducted a review of existing literature in this field, which yielded very limited empirical data. One review by Reiter-Palmon et al.^30^ stands out for its examination of virtual teams, including an exploration of creativity within these settings. Despite the depth of their review, they highlighted a significant shortfall in empirical studies, noting that much of the existing discourse is theoretical and lacks rigorous evaluation of the factors that might either facilitate or inhibit creativity in virtual environments.

Among the few empirical studies available, Han et al. offered some insights^31^. This study compared various communication platforms—VC, voice-only calls, text-only interactions, and traditional FTF meetings—to assess their impact on the quality and creativity of ideas generated by groups. However, given that this research was conducted over a decade ago, it is important to consider the technological limitations of VC at that time. Han et al. concluded that there were no significant differences in the quality or creativity of ideas generated across the different platforms, but this finding may warrant re-examination in light of advancements in VC technology.

In a recent study^32^, researchers conducted a series of experiments to scrutinize the differential impacts of VC and FTF interactions on creativity and decision-making. These experiments specifically focused on brainstorming activities undertaken by pairs of participants. Initial findings revealed that, while couples engaged in video calls generated a quantitatively reduced set of ideas compared to their in-person counterparts, the ideas they did produce were qualitatively superior, as evidenced by higher creativity scores and lower decision error rates. To delve deeper into these results, the researchers formulated a hypothesis positing that the constrained visual field in VC could be a limiting factor for idea generation. The hypothesis suggests that the bounded space shared by couples narrows the visual field, and this in turn restricts cognitive scope. Subsequent experiments validated this hypothesis. Couples participating via video calls were observed to focus more intently on their screens, thereby narrowing their visual field. This restricted gaze was not only limited to their partner on the screen but also led to reduced awareness of their physical surroundings. Quantitatively, this was reflected in lower recall scores for objects present in the room. Importantly, these room gaze and object recall metrics were found to be significantly correlated with the number of creative ideas generated, thereby substantiating the hypothesis that a narrowed visual focus could indeed be a cognitive bottleneck.

To reinforce the robustness of these findings, the researchers replicated the experiment across five distinct locations with a larger sample size. The results were consistent: VC was associated with fewer generated ideas but higher decision quality. However, it is worth noting that the positive impact on decision quality was somewhat mitigated when controlling for the number of ideas generated, suggesting a relationship between the medium of interaction and cognitive outcomes.

The primary objective of our research is to address some of the many gaps in the literature and compare the effects of VR, and FTF interactions on a number of psychological constructs. In the first phase, participants are tasked with generating as many ideas as possible on an assigned problem, utilizing divergent thinking, namely a thought process used to generate creative ideas by exploring many possible solutions^33^. This 20-min phase allowed us an evaluation of creativity, measured by the number of ideas generated and assessed across three dimensions: uncommonness, remoteness, and cleverness^34^. Drawing on prior research, we hypothesize that VR immersion can lead to levels of creativity higher than the other two conditions^25^, or comparable to those of FTF encounters^29^, in terms of the number of ideas generated.

In the second phase, participants are instructed to collaboratively rank the top 5 ideas generated. This 20-min, timed phase employs a methodology adapted from previous research^29^ to assess group decision-making during convergent thinking. The level of decision-making is calculated by dividing the time spent by the number of ideas generated. Our hypothesis posits no significant differences in decision-making across the three settings^24^, although video calls may yield distinct outcomes compared to FTF interactions^32^.

While interaction dynamics have yet to be fully explored, existing studies suggest that VR offers advantages such as enhanced social presence^26^ and a more focused, relaxed environment^28^. We hypothesize that these factors may positively influence interaction, leading to increased collaboration and reduced conflict. To assess this, all sessions were video recorded and later analyzed using the Bales Interaction Process Analysis evaluation grid^35^.

Given that most participants in this study were using VR for the first time, we anticipated that the novelty may result in perceived difficulty and potentially greater fatigue. To explore this, participants completed the Zoom Exhaustion and Fatigue Scale^36^.

Furthermore, given that VR elicits emotional responses and presence comparable to the physical world^37^, we expected that the immersive nature of VR might impact flow, a psychological state characterized by complete absorption and engagement in an activity, leading to enhanced performance and well-being^28^. Studying flow in this context is particularly relevant as it can offer insights into how different interaction modalities may influence cognitive engagement and task performance. This is measured using the Flow Short Scale^38^. Additional questionnaires assess participants' perceived performance using the Perceived Performance Scale^39^ and their identification with their group via the Single-Item Social Identification^40^.

In summary, our research presents several hypotheses informed by the different impacts of interaction modalities—VR, VC, and FTF—on some psychological constructs of individuals in group dynamics. We hypothesize that VR immersion will foster higher creativity levels and flow compared to VC and potentially match FTF in terms of idea generation, based on its immersive and stimulating nature. For decision-making efficacy, we anticipate no significant differences across the modalities, acknowledging the complexity of cognitive processes in diverse settings. Additionally, we hypothesize that VR will enhance collaboration and reduce conflict due to its unique spatial and social dynamics. Lastly, we expect the novelty of VR to introduce an element of perceived difficulty, potentially leading to greater fatigue among first-time users.

## Results

In this study, we examined the impact of different interaction settings on decision-making and creativity. A total of 56 participants were randomly assigned to one of three conditions: FTF, VC, and VR. Each group engaged in a 20-min brainstorming session to generate ideas on an assigned problem, followed by a 20-min phase to collaboratively rank the top 5 ideas. Creativity was assessed using three dimensions: uncommonness, remoteness, and cleverness. Decision-making efficacy was calculated by dividing the time spent on ranking by the number of ideas generated. Participants also completed 4 questionnaires: the Zoom Exhaustion and Fatigue Scale, the Flow Short Scale, the Perceived Performance Scale, and the Single-Item Social Identification.

All statistical analyses were conducted using R software (version 4.2.3) and Microsoft Excel. We began with a Shapiro–Wilk Normality Test to assess data distribution across experimental conditions (VC, VR, and FTF), guiding our selection of appropriate statistical tests. For normally distributed data, we applied a repeated-measures Analysis of Variance (ANOVA) with a significance threshold of *p* < 0.05, adjusted for multiple comparisons using False Discovery Rate (FDR) correction. Post-hoc analyses for significant findings involved *t*-tests for dependent samples across condition pairs (VC-VR, VR-FTF, and VC-FTF). In cases of non-parametric data distributions, we used the Friedman test and Wilcoxon Signed-Rank Test for paired comparisons, with FDR correction applied to p-values for significance determination. Additionally, Pearson correlation and regression analyses explored the relationship between participants' age and their questionnaire responses, considering *p*-values less than 0.05 as statistically significant. See the Data Analysis section below for more details.

**Figure 1:**
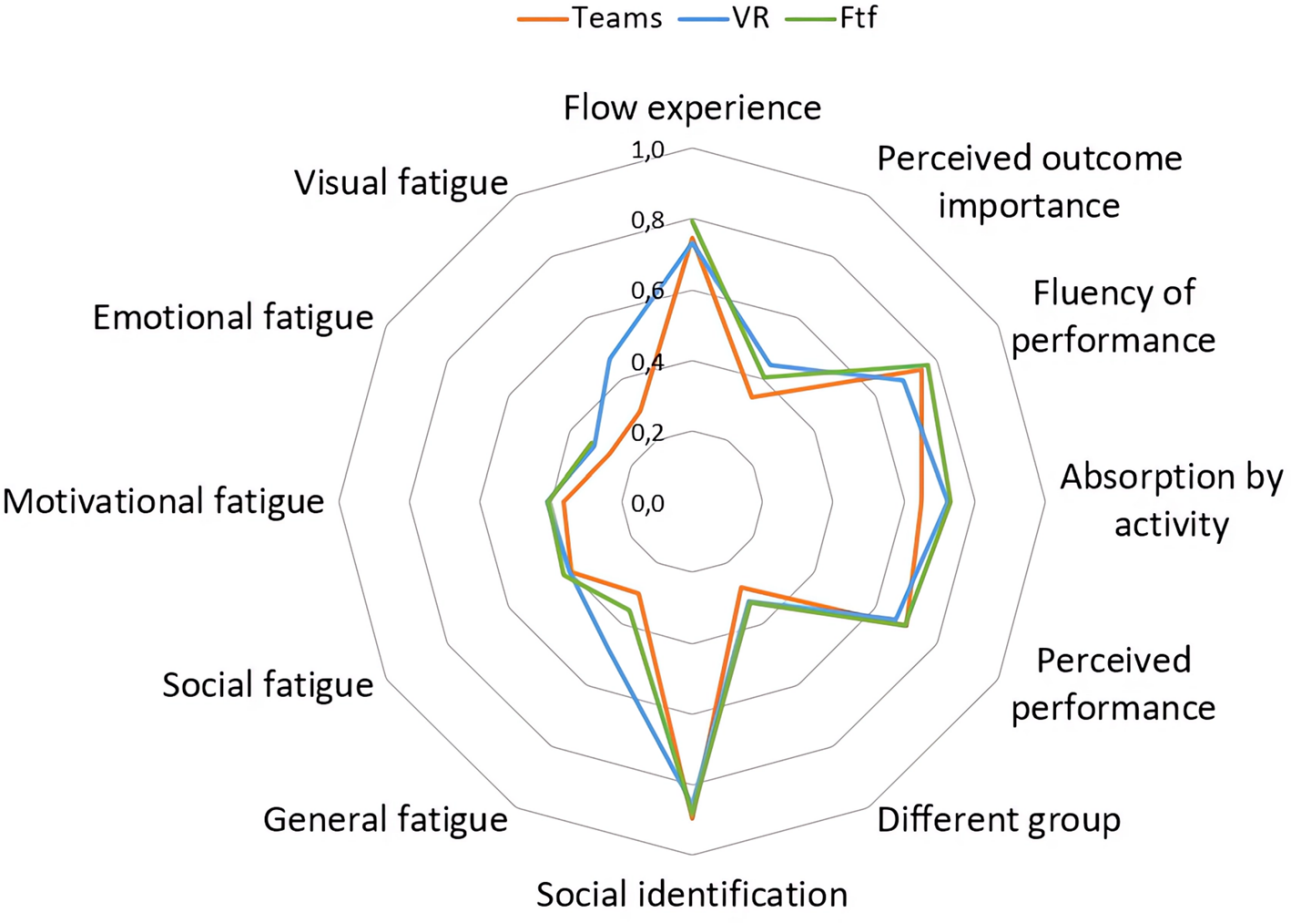
Multidimensional analysis of psychological and cognitive metrics across interaction settings. This figure graphically represents the mean scores for each of the three interaction conditions—face-to-face (FTF, represented by the green line), video call via Teams (represented by the red line), and virtual reality (VR, represented by the blue line)—across various psychological and cognitive constructs. The constructs included are Flow experience, Perceived Performance, Social Identification, and Zoom Exhaustion and Fatigue Scale. Data normalization was applied to account for different measurement scales across the constructs.

**Figure 2:**
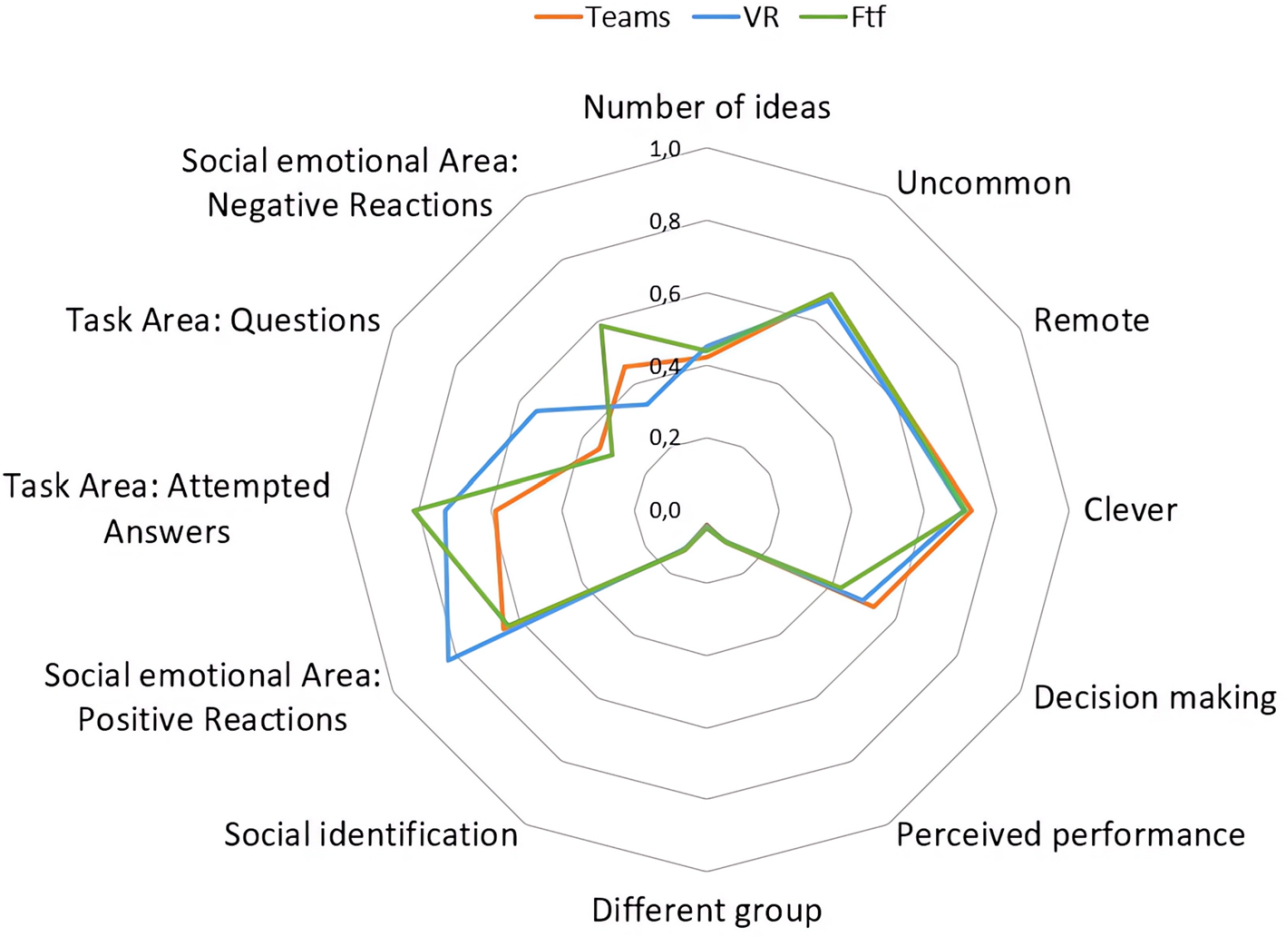
Comparative analysis of creativity, decision-making, and interaction dynamic. This figure illustrates the mean scores for each interaction condition—face-to-face (FTF, represented by the green line), video call via Teams (represented by the red line), and virtual reality (VR, represented by the blue line)—in relation to creativity, decision-making, and the four quadrants assessed by the Bales Interaction Process Analysis grid^35^. Data normalization was performed to ensure comparability across variables that were measured on different scales.

As depicted in Figs. 1 and 2, the graphs provide a comprehensive visualization of the outcomes across all investigated variables, which are denoted in the respective corners of the radar plot. To facilitate cross-variable comparisons, data normalization was employed, given that the constructs under investigation were assessed using disparate measurement scales. For variables lacking a predefined scale—such as the number of ideas generated—the normalization was executed based on the maximum observed value for that specific variable.

Figure 1 summarizes the outcomes from questionnaires focused on Flow experience, perceived performance, social identification, and the Zoom Exhaustion and Fatigue Scale. Conversely, Fig. 2 encompasses the analyses pertinent to creativity, decision-making, and the four dimensions scrutinized via the Bales Interaction Process Analysis grid^35^.

Table 1 presents the means and standard deviations for each dimension across the different types of interaction settings. Tables 2 and 3 delineate the outcomes of the statistical analyses conducted. Specifically, Table 2 contains the results for data samples conforming to a normal distribution, for which Analysis of Variance (ANOVA) and t-tests were employed. Conversely, Table 3 features the results for non-parametric distributions, analyzed using Friedman and Wilcoxon tests. In both tables, the significance level of the p-value is denoted by asterisks.

**Table 1:** Descriptive statistics for interaction conditions.

Variables	Video conference		Virtual reality		Face-to-face	
	M	SD	M	SD	M	SD
FSS						
Flow experience	5.21	0.86	5.14	1.07	5.55	0.70
Perceived outcome importance	2.38	0.88	3.12	1.14	2.85	1.14
Fluency of performance	5.24	1.05	4.83	1.34	5.40	0.94
Absorption by activity	4.55	0.88	5.05	0.95	5.12	0.68
ZEFS						
General	1.50	0.74	2.39	1.11	1.76	0.73
Social	1.97	0.96	2.00	1.05	2.09	1.09
Motivational	1.83	0.89	2.06	1.01	2.04	1.04
Emotional	1.35	0.76	1.59	0.75	1.64	0.82
Visual	1.49	0.65	2.33	1.02		
PPS						
Perceived performance	4.91	1.05	4.67	1.3	4.88	1.25
Different group	1.97	1.08	2.27	1.57	2.29	1.45
SISI						
Single-item social identification	6.26	1.07	6.00	1.21	6.18	0.94
Number of ideas	15.60	5.10	16.8	7.77	16.2	5.03
Evaluation of ideas						
Uncommon	3.44	0.21	3.35	0.30	3.44	0.28
Remote	3.05	0.36	2.99	0.26	3.06	0.31
Clever	3.66	0.25	3.55	0.14	3.57	0.13
Decision making	40.94	16.64	38.44	15.62	32.85	16.29
IPA						
Social emotional area: positive reactions	76.90	16.18	97.40	22.55	75.00	9.71
Task area: attempted answers	82.10	12.82	101.3	15.55	113.6	14.78
Task area: questions	15.00	8.58	24.00	10.02	13.40	2.95
Social emotional area: negative reactions	10.90	5.93	8.00	4.29	14.10	5.92

This table presents the means and standard deviations for all measured dimensions, segmented by each type of interaction condition: face-to-face, video conference and virtual reality. M stand for mean, and SD for standard deviation.

**Table 2:** Parametric statistical analysis results.

Variables	VC × VR × FTF		VC × VR			VR × FTF			VC × FTF		
	F	P	M diff	T	P	M diff	T	P	M diff	T	P
FSS											
Perceived outcome importance	7.781	0.0008***	− 0.71	− 4.343	0.0001***	0.36	1.288	0.2055	− 0.35	− 2.458	0.0186*
Fluency of performance	3.804	0.0266*	0.48	1.551	0.1291	− 0.60	− 2.574	0.0141*	− 0.12	− 1.213	0.2326
Absorption by activity	7.147	0.0014**	− 0.49	− 2.480	0.0177*	0.05	− 0.432	0.6682	− 0.44	− 4.837	2.207e−05***
PPS											
Perceived performance	0.776	0.4640	0.24	1.062	0.2951	− 0.21	− 1.108	0.2747	0.02	0.118	0.9070
Creativity											
Number of ideas	0.123	0.8850	− 1.20	− 0.513	0.6200	0.60	0.199	0.8463	− 0.60	− 0.347	0.7362
Decision making	1.038	0.3750	2.51	0.344	0.7389	5.59	1.063	0.3156	8.10	1.882	0.0926
IPA											
Social emotional area: positive reactions	4.757	0.0219*	− 20.50	− 2.148	0.0603	22.40	2.672	0.0255*	1.90	0.330	0.7491
Task area: attempted answers	18.380	4.49e−05***	− 19.20	− 3.191	0.0110*	− 12.30	− 2.317	0.0457*	− 31.50	− 7.442	3.926e−05***
Social emotional area: negative reactions	3.116	0.0689	2.90	1.174	0.2704	− 6.10	− 2.690	0.0248*	− 3.20	− 1.238	0.2471

This table displays the outcomes of parametric statistical analyses, including ANOVA and t-tests, conducted on data samples with a normal distribution. Significance levels are indicated by asterisks next to the *p*-values. False Discovery Rate (FDR) correction was applied for multiple comparisons. VC stands for Video Conference, VR for Virtual Reality, and FTF for Face To Face interactions. M diff stands for Mean Difference.

**Table 3:** Non-parametric statistical analysis results.

Variables	VC × VR × FTF		VC × VR			VR × FTF			VC × FTF		
	Chi-squared	*P*	M diff	V	*P*	M diff	V	*P*	M diff	V	*P*
FSS											
Flow experience	2.430	0.2968	0.13	391.5	0.7662	− 0.38	203.5	0.0260*	− 0.25	168.5	0.0099**
ZEFS											
General	26.922	1.426e−06***	− 0.89	73.5	0.0002***	0.63	444.0	0.0035**	− 0.26	70.5	0.0076**
Social	2.346	0.3095	− 0.03	149.0	0.9885	− 0.09	165.0	0.5715	− 0.12	152.0	0.5570
Motivational	1.863	0.3939	− 0.23	160.0	0.2156	0.02	203.5	1.0000	− 0.21	124.5	0.1201
Emotional	6.615	0.0366*	− 0.24	60.0	0.0955	− 0.05	124.5	0.6906	− 0.29	50.0	0.0132*
Visual			− 0.84	66.5	0.0002***						
PPS											
Different group	0.640	0.7264	− 0.30	141.5	0.5792	− 0.02	123.0	0.9218	− 0.32	99.5	0.2449
SISI											
Single-item social identification	2.355	0.3081	0.26	98.0	0.1163	− 0.18	46.5	0.4445	0.08	48.0	0.4644
Evaluation of ideas											
Uncommon	1.800	0.4066	0.09	20.0	0.3525	− 0.09	8.0	0.3525	0	16.0	0.8336
Remote	0.200	0.9048	0.06	16.0	0.7998	− 0.07	10.0	0.5541	− 0.01	17.0	0.9442
Clever	2.600	0.2725	0.11	21.0	0.2719	− 0.02	13.0	0.9326	0.09	28.0	0.1834
IPA											
Task area: questions	15.436	0.0004***	− 9.00	0	0.0058**	10.60	55.0	0.0059**	1.60	24.0	0.9055

This table showcases the results of non-parametric statistical analyses, specifically Friedman and Wilcoxon tests, conducted on data samples with non-normal distributions. Significance levels are denoted by asterisks adjacent to the *p*-values. False Discovery Rate (FDR) correction was applied for multiple comparisons. VC stands for Video Conference, VR for Virtual Reality, and FTF for Face To Face interactions. M diff stands for Mean Difference.

### Flow short scale

The analysis yielded several noteworthy findings concerning the perception of the flow state across different interaction conditions.

#### Flow experience

The Flow Experience refers to the set of elements that characterize the sensation of the state of flow, such as the sense of absorption in the activity, the right level of challenge, the lack of perception of time passing, and the spontaneity of thoughts and actions. Statistically significant differences were observed in the perception of flow between VR and FTF conditions (*p* = 0.0260*; Mean Difference = − 0.38), as well as between VC and FTF conditions (*p* = 0.0099**; Mean Difference = − 0.25). The FTF condition registered the highest mean score, while the VR condition recorded the lowest.

#### Perceived outcome importance

The Perceived Outcome Importance reflects the participant's perception of achieving a good result and the significance of the activity. In these terms, the VR condition yielded the highest mean, whereas the VC condition had the lowest. A one-way ANOVA revealed a highly significant *p*-value of 0.0008***. Subsequent pairwise t-tests between VC and FTF (*p* = 0.0186*; Mean Difference = − 0.35) and VC and VR (*p* = 0.0001***; Mean Difference = − 0.71) were also significant.

#### Fluency of performance

The fluidity of performance assesses the participant's perception of the spontaneity of their thoughts and actions, engaging in an activity seamlessly and without interruptions. For this dimension, a significant ANOVA *p*-value of 0.0266* was obtained. The lowest mean was associated with the VR condition, while the highest was observed in the FTF condition. A significant t-test result was found specifically for this pair (*p* = 0.0141*; Mean Difference = − 0.60).

#### Absorption by activity

The Absorption by Activity indicates the sense of absorption in participating in the activity. In this case, the VC condition exhibited the lowest mean, while the FTF condition had the highest. The ANOVA yielded a significant *p*-value of 0.0014. Pairwise *t*-tests revealed significant *p*-values for VC versus VR (*p* = 0.0177*; Mean Difference = − 0.49) and VC versus FTF (*p* < 0.0001***; Mean Difference = − 0.44).

### Zoom exhaustion and fatigue scale (ZEF scale)

Non-parametric statistical methods were employed for all dimensions of the ZEFS questionnaire.

#### General fatigue

The first dimension of the Friedman test is General Fatigue, that in the ZEFS questionnaire refers to the general experience of being tired. The test yielded highly significant results for the dimension of General Fatigue (*p* < 0.0001***). Subsequent Wilcoxon tests for pairwise comparisons revealed significant *p*-values across all three conditions, thereby establishing the means as significantly distinct. The ranking order of the means is as follows: VC (M = 1.50), FTF (M = 1.76), and VR (M = 2.39). The pairwise results are: VC versus VR (*p* = 0.0002***; Mean Difference = − 0.89), VR versus FTF (*p* = 0.0035**; Mean Difference = 0.63), and VC versus FTF (*p* = 0.0076**; Mean Difference = − 0.26).

#### Emotional fatigue

Another dimension that yielded a significant Friedman test *p*-value was Emotional Fatigue (*p* = 0.0366*), which is defined as the state of feeling overwhelmed, drained and used up. In this dimension, the only pairwise comparison that reached statistical significance in the Wilcoxon test was between VC and FTF (*p* = 0.0132*; Mean Difference = − 0.29). The FTF condition registered the highest mean (M = 1.64), whereas the VC condition had the lowest (M = 1.35).

#### Visual fatigue

The dimension of Visual Fatigue, defined as any subjective visual symptom or distress resulting from use of one’s eyes, was assessed solely in the VC (M = 1.49) and VR (M = 2.33) conditions. A Wilcoxon test revealed a highly significant *p*-value for this pairwise comparison (*p* = 0.0002***; Mean Difference = − 0.84).

### Perceived performance scale (PPS)

#### Perceived performance

The initial dimension explored by the PPS was Perceived Performance. It refers to the perception of having completed the task well, of having generated a large number of ideas and that these are of good quality. The ANOVA analysis yielded a non-significant *p*-value (*p* = 0.4640), suggesting that participants, on average, did not perceive any performance disparities across the three interaction conditions. Subsequent t-tests corroborated this finding, as they also produced non-significant *p*-values.

#### Different group

The second dimension, termed Different Group, aimed to assess whether participants believed they would generate either a greater number or higher quality of ideas with alternative group members. For this dimension, a Friedman test was conducted, resulting in a non-significant *p*-value (*p* = 0.7264). Pairwise comparisons using the Wilcoxon test further substantiated this outcome, as all returned non-significant *p*-values.

### Single-item social identification (SISI)

This questionnaire consists of a single item and investigates the perception of belonging to the group. The Friedman test conducted on the data of the three conditions obtained a non-significant *p*-value (*p* = 0.3081). Further comparisons between pairs using the Wilcoxon test also all reported non-significant *p*-values.

### Interaction process analysis (IPA)

This analytical tool was employed to scrutinize all interactions across the different conditions. Each interaction was categorized into distinct areas of interaction, and several significant findings were observed. These are elaborated upon below.

#### Social-emotional area: positive reactions

ANOVA analysis for this area yielded a significant *p*-value (*p* = 0.0219*). The condition with the highest mean frequency of positive interactions was VR (M = 97.4), while the FTF condition registered the lowest mean (M = 75). Pairwise *t*-tests revealed a trend towards significance for the VC × VR comparison (*p* = 0.0603; M diff = − 20.5) and a significant *p*-value for the VR × FTF comparison (*p* = 0.0255*; M diff = 22.40).

#### Task area: attempted answers

This area encompasses all participant attempts at providing answers and explanations. The ANOVA analysis yielded a highly significant *p*-value (*p* = 4.49e−05***). Subsequent t-tests for all pairwise comparisons were also significant: VC × VR (*p* = 0.0110*; M diff = − 19.20), VR × FTF (*p* = 0.0457*; M diff = − 12.30), and VC × FTF (*p* = 3.926e−05***; M diff = − 31.50). Given the significant differences across all pairs, the conditions can be ranked by their respective means: VC (M = 82.1), VR (M = 101.3), and FTF (M = 113.6).

#### Task area: questions

This area includes all attempts to ask questions and requests for clarification. It exhibited a non-parametric distribution and was thus analyzed using Friedman and Wilcoxon tests. The overall comparison yielded a highly significant *p*-value (*p* = 0.0004***). Pairwise comparisons revealed significant *p*-values for VC × VR (*p* = 0.0058**; M diff = − 9.00) and VR × FTF (*p* = 0.0059**; M diff = 10.60). Given these results and the observed means (VC M = 15; VR M = 24; FTF M = 13.4), it can be concluded that VR significantly outpaces the other conditions in terms of the frequency of questions and requests for clarification.

#### Social-emotional area: negative reactions

This area includes interactions that generate tension, attempts to dominate, and rejection of others' ideas. The ANOVA analysis produced a *p*-value approaching significance (*p* = 0.0689). Subsequent *t*-tests revealed a significant *p*-value only for the VR × FTF pair (*p* = 0.0248*; M diff = − 6.10). VR registered the lowest mean frequency of negative interactions (M = 8), while FTF had the highest (M = 14.1).

### Creativity

#### Preliminary analysis: topic variability

Prior to assessing the influence of communication settings on creativity, an initial analysis was conducted to evaluate the potential variability in the number of ideas generated across three different thematic areas (see Methods)—namely Tourism (M = 13.6), Restaurant (M = 19.3), and Pollution (M = 15.7). Shapiro's test confirmed the normality of the distributions for these samples. A repeated-measures ANOVA yielded a significant *p*-value (*p* = 0.0344*). Pairwise t-tests revealed a significant difference between the Tourism and Restaurant topics (*p* = 0.0301*; M diff = − 5.7). However, this variability was deemed inconsequential for the broader study, as each topic was employed in a balanced manner across all conditions.

#### Number of ideas generated

The first dimension of creativity assessment taken into consideration was the average number of ideas generated for each condition, where a significantly higher number would have indicated greater creativity in generating ideas. The mean number of ideas generated for each experimental condition were as follows: VC (M = 15.6), VR (M = 16.8), and FTF (M = 16.2). Given the normal distribution of these data, an ANOVA test was employed for comparative analysis, resulting in a non-significant difference (*p* = 0.8850).

#### Qualitative analysis: OSF tool

Subsequently, two independent human raters evaluated each generated idea using the OSF tool, which employs three distinct criteria: Uncommon, Remote, and Clever. The 'uncommon' dimension measures how rare a single idea is among the collected ideas, while the 'remote' dimension gauges how far that idea deviates from common ones typically associated with that topic. The last dimension, 'clever,' pertains to how much that idea is perceived as smart, comprehensive, and well-articulated. The choice of these three dimensions is in in line with systematic frameworks for divergent thinking test scoring as outlined in the psychological literature on creativity^34^.The mean ratings for each criterion across the conditions were: Uncommon (VC M = 3.44; VR M = 3.35; FTF M = 3.44), Remote (VC M = 3.05; VR M = 2.99; FTF M = 3.06), and Clever (VC M = 3.66; VR M = 3.55; FTF M = 3.57).

Given the non-parametric nature of these samples, Friedman tests were conducted for comparative analysis. The results indicated non-significant *p*-values across all three dimensions: Uncommon (*p* = 0.4066), Remote (*p* = 0.9048), and Clever (*p* = 0.2725).

A secondary, additional analysis was conducted to assess the concordance between evaluators' ratings. Pearson's analysis was used for comparing the average evaluation criteria scores for each group, as determined by researchers. This analysis yielded r-values ranging from 0 to 1, with higher scores indicating greater concordance between ratings. A threshold value typically considered indicative of good concordance is a score greater than 0.7. The following results were obtained:

**Table:** 

Variables	Uncommon	Remote	Clever
Teams	0.4308	0.8934*	0.8832*
VR	0.5990	0.8318*	0.1691
FTF	0.4852	0.6843	0.4132

The results indicate good concordance for values related to the Remote, a lack of significant concordance for the Uncommon variable, and a value above 0.7 only in the Teams condition for Clever.

### Decision making

To assess the impact of the experimental settings on group decision-making efficacy, we computed a ratio representing the time required to reach a consensus on idea ranking relative to the number of ideas generated in each session. This metric reflects how groups navigate the balance between idea generation and evaluation, a key component of collaborative decision-making. In comparing different communication modes (VR, FTF, VC), it provides insights into how various mediums impact the group's ability to efficiently process and prioritize ideas, thereby influencing overall team productivity and cognitive load. The mean ratios for the three experimental conditions were as follows: VC (M = 40.94), VR (M = 38.44), and FTF (M = 32.85). An Analysis of Variance (ANOVA) was conducted to compare these means, yielding a non-significant *p*-value (*p* = 0.3750).

### Correlation and linear regression

Table 4 presents the outcomes of correlation analyses, along with associated *p*-values, examining the relationship between participant age and responses across all questionnaires within the various experimental conditions. With respect to the questionnaire probing the state of flow, the correlation analyses between its multiple constructs and participant age yielded no statistically significant results.

**Table 4:** This table presents the outcomes of correlation analyses between participant age and responses to various questionnaires administered across different experimental conditions.

Variables	VC			VR			FTF		
	r	*F*	*P*	r	*F*	*P*	r	*F*	*P*
ZEFS									
General	− 0.2725	2.9682	0.0933	− 0.1871	1.3428	0.2540	− 0.4263	8.2191	0.0068**
Social	− 0.2087	1.6850	0.2023	− 0.1761	1.1844	0.2835	− 0.0822	0.2517	0.6189
Motivational	− 0.2812	3.1771	0.0829	− 0.3406	4.8542	0.0339*	0.0163	0.0099	0.9215
Emotional	− 0.1447	0.7918	0.3793	− 0.3525	5.2483	0.0278*	− 0.1672	1.0645	0.3089
Visual	− 0.2835	3.0588	0.0891	− 0.1301	0.6022	0.4430			
PPS									
Perceived performance	− 0.1300	0.6356	0.4304	0.0398	0.0588	0.8098	− 0.2174	1.8347	0.1838
Different group	− 0.0773	0.2223	0.6401	− 0.3766	6.1143	0.0181*	− 0.2819	3.1948	0.0821

Each cell contains the correlation coefficient and associated *p*-value, providing a statistical measure of the strength and direction of the relationship between age and questionnaire responses. False Discovery Rate (FDR) correction was applied for multiple comparisons. VC stands for Video Conference, VR for Virtual Reality, and FTF for Face To Face interactions, ZEFS stands for Zoom Exhaustion and Fatigue Scale, and PPS stands for Perceived Performance Scale.

In the analysis of responses to the Zoom Exhaustion and Fatigue Scale (ZEFS), several noteworthy correlations with age emerged. In the VC condition, three dimensions—General Fatigue (r = − 0.2725; *p* = 0.0933), Motivational Fatigue (r = − 0.2812; *p* = 0.0829), and Visual Fatigue (r = − 0.2835; *p* = 0.0891)—displayed p-values approaching significance, all indicating a negative correlation with age.

For the VR condition, significant p-values were observed in the dimensions of Motivational Fatigue (r = − 0.3406; *p* = 0.0339*) and Emotional Fatigue (r = − 0.3525; *p* = 0.0278*). Both dimensions exhibited a negative correlation, suggesting that as participant age increased, reported fatigue levels decreased.

In the FTF condition, a significant negative correlation was found for the dimension of General Fatigue (r = − 0.4263; *p* = 0.0068**).

Lastly, within the context of the Perceived Performance Scale (PPS), a significant p-value emerged in the linear regression analysis between age and the "Different Group" dimension, but only in the VR condition (r = − 0.3766; *p* = 0.0181*).

## Discussion

In the present study we evaluated the differential impacts of 3 distinct modes of interaction—FTF, VC, and VR—on group work dynamics. Specifically, the study focused on variables such as idea generation, decision-making, and various psychological constructs.

Our data revealed that FTF interactions significantly outperformed the other two modes in enhancing components of the state of flow, including flow experience, fluency of performance, and activity absorption. Interestingly, despite VC being second in promoting flow experience, VR exhibited a lower average but a higher standard deviation across all constructs. This variability suggests that the VR experience is highly individualized, particularly among users who are unfamiliar with the technology.

The environmental context had a significant impact on activity absorption, a key component of the Flow State Scale (FSS). Video recordings of VC sessions revealed frequent instances of participants engaging with their smartphones, thereby diluting their focus on the task at hand. Such distractions were notably less prevalent in FTF interactions and were obviously non-existent in VR settings due to the immersive nature of the technology. Consequently, VR and FTF modalities yielded comparable levels of activity absorption, underscoring the potential of VR to simulate the cognitive engagement typically associated with physical presence.

The VR setting was associated with higher scores in the construct of perceived importance of the task outcome. This could be attributed to the novelty of the VR experience, which may have heightened participants' sense of commitment^41^. In contrast, VC sessions scored lower in perceived importance. This could be attributed to the ubiquity of VC in contemporary work settings; indeed, 87.5% of participants reported engaging in more than eight video calls per week. Furthermore, the informal, often domestic settings in which VC sessions occurred may have attenuated the social pressures that typically enhance task commitment.

Drawing upon the findings from the Flow State Scale (FSS), FTF interactions appear to be the most effective modality for fostering both task importance and activity absorption^42^. However, when such interactions are not feasible, VR emerges as a viable alternative, offering comparable levels of activity absorption and a heightened sense of task importance.

Analysis of the ZEF (Zoom Exhaustion Fatigue) questionnaire revealed that VR was the most cognitively taxing of the 3 interaction modalities, while VC was the least fatiguing. Responses spanned the entire range of the Likert scale, underscoring the subjective nature of the experience across participants^43^. Notably, both VR and FTF interactions necessitated physical presence in company offices, thereby imposing additional cognitive and physical demands on participants as in dual tasks^44^.

Two additional dimensions of fatigue emerged from the ZEF questionnaire: visual fatigue and emotional fatigue. VR was associated with higher visual fatigue, likely exacerbated by participants' unfamiliarity with the headsets. While our study did not directly evaluate cybersickness, its potential influence, particularly of visual fatigue, warrants consideration. Cybersickness can manifest as ocular discomfort, among other symptoms, possibly contributing to visual fatigue observed in participants. Acknowledging this, future research could explicitly examine the role of cybersickness and its impact on visual and cognitive performance in virtual environments^45^. On the other hand, FTF interactions were linked to elevated levels of emotional fatigue, potentially due to the direct social and emotional engagement required in such settings.

Bales Interaction Process Analysis (IPA) model yielded intriguing insights into the socio-emotional dynamics of group interactions. VR outperformed FTF settings in fostering positive socio-emotional behaviors, such as mutual esteem, support, and understanding. Moreover, VR was associated with fewer negative socio-emotional behaviors, suggesting that it may cultivate a more harmonious group environment^35^.

In terms of task-oriented behaviors, FTF interactions yielded the highest frequency of response attempts, including proposed ideas and clarifications. VR followed closely, significantly outperforming VC in this regard. Furthermore, VR sessions were characterized by a higher frequency of questions, indicating a greater willingness among participants to seek clarification and share ideas^35^.

Unexpected negative correlations emerged between age and various constructs, including motivational fatigue and emotional fatigue in VR, and general fatigue in FTF settings. These findings suggest that increased familiarity and experience with different interaction modalities may mitigate the perception of fatigue as one ages.

Our study elucidates the effects of different interaction modalities—FTF, VR, and VC—on group dynamics, cognitive load, and task performance. VR, although a nascent technology with which participants had limited familiarity, emerged as a medium for fostering a peaceful and collaborative environment. This observation is substantiated by higher scores in the positive socio-emotional domain as per Bales Interaction Process Analysis (IPA) model^35^. Interestingly, this modality also elicited a greater perception of the task's importance, potentially due to the immersive nature of the technology that commands heightened attention and commitment^46^.

However, the VR condition was not without its drawbacks. Participants reported elevated levels of general and visual fatigue, likely exacerbated by their inexperience with the technology. This aligns with existing literature highlighting the cognitive and physiological demands of virtual environments^2^. Conversely, FTF interactions were found to be most conducive for the generation of ideas, corroborated by higher scores in flow experience and activity absorption metrics. This is consistent with theories positing that physical presence enhances cognitive engagement and social cues, thereby facilitating a more effective collaborative process^47^.

VC, the most ubiquitous form of remote interaction, was the least cognitively taxing but also the least engaging, as evidenced by lower scores in activity absorption and the frequency of response attempts. This could be attributed to the familiarity and ubiquity of the technology. This familiarity may have attenuated the perceived importance of the task and the social pressures that might otherwise enhance engagement.

Our analysis also revealed intriguing age-related trends. Younger participants reported higher levels of motivational and emotional fatigue in the VR condition, suggesting either heightened sensitivity or perhaps challenges in emotion regulation. In contrast, older participants, who presumably have more experience with FTF meetings, reported lower levels of general fatigue in that condition. These findings hint at the complex interplay between age, experience, and the cognitive and emotional demands of different interaction modalities.

Concerning our explicit expectations, the hypothesis that VR would enhance creativity was partially supported, with VR showing promise in specific creative aspects but not uniformly outperforming other modes. Here, creativity assessments were conducted by two trained raters, adhering to specific criteria to ensure consistency^34^. Given the subjective nature of creativity evaluation, some variability in scoring was expected (only for some of the measures there was good consistency), as this reflects the complex, multifaceted nature of creative thinking.

Our prediction of decision-making efficacy being similar across VR, VC, and FTF was largely confirmed, indicating the complexity of these cognitive processes in different settings. The anticipated improvement in collaboration and reduction of conflict in VR was observed to some extent. Lastly, the hypothesis regarding VR's novelty leading to greater fatigue was substantiated, especially among first-time users.

### Limitations and future work

While the present study garnered substantial data from a large participant pool, yielding significant insights into the dynamics of different interaction modalities, it is important to acknowledge its limitations and suggest directions for future research.

One primary constraint pertains to the potential for experimenter bias in the analysis of video recordings. Despite the utilization of a manual designed to objectify the categorization of various interventions, the fact that the recordings were evaluated by the study's designers introduces the possibility of confirmation bias. A methodological refinement to mitigate this limitation would be to employ multiple independent raters, although the time-intensive nature of this task rendered it impractical for the current study.

A second limitation arises from the inability to randomize the sequence of experimental conditions fully. The logistical necessity of conducting the VR and FTF sessions consecutively—aimed at optimizing the researchers' travel to corporate locations—may have introduced an order effect that could influence the results.

Additionally, the nascent state of VR technology itself serves as a limitation. While advancements in hardware are likely to yield more user-friendly and less fatiguing devices, the current study had to contend with the limitations of beta-phase software (Horizon Workrooms), which occasionally required participants to recalibrate settings, thereby potentially affecting their responses and task performance.

Lastly, the study was designed with a focus on non-expert users of VR technology, to reflect real-world scenarios where professionals often encounter new technologies without prior training. Future research could profitably extend this inquiry to include participants with varying degrees of familiarity with VR, thereby offering a more nuanced understanding of how prior experience with the technology might modulate the observed effects across different cognitive and emotional constructs.

## Conclusion

In conclusion, this study provides a preliminary analysis of the effects of VR, VC, and FTF modalities on group dynamics and individual psychological constructs within professional settings. Our findings reveal that each modality offers benefits and limitations, impacting aspects like creativity, decision-making, flow experience, and fatigue in distinct ways. While FTF interactions remain the most effective in fostering flow, VR emerges as a promising alternative, offering immersive experiences that enhance task importance, more positive emotions and collaboration. However, the technology's novelty can lead to increased fatigue, underscoring the need for further ergonomic advancements and user acclimation. VC, widely used in modern workplaces, presents a less demanding but also less engaging option. These insights are crucial for organizations navigating the evolving landscape of digital collaboration, providing a foundation for future research to optimize the use of these modalities in enhancing workplace productivity and creativity.

## Methods

### Participants

The study’s participant pool was drawn from Terna S.p.A., a leading Italian electricity transmission company. Recruitment was facilitated by organizational managers to ensure the formation of heterogeneous yet familiar work groups, thereby isolating the influence of the experimental setting from the variable of unfamiliarity among participants. The initial cohort comprised 56 individuals, organized into 12 groups of four members each. During data collection some participants did not participate in all sessions, therefore their data and those of their groups were excluded. The final sample was made up of 40 people, divided into 10 work groups. The mean age of the participants was 38.03 years, with a standard deviation of 10.9 and an age range of 25–62 years. The gender distribution included 22 males and 18 females. A preliminary questionnaire assessed the frequency of video call usage for work-related communication, employing a Likert scale ranging from “0–1” to “8 or more” times per week. Notably, 87.5% of participants reported engaging in “8 or more” video calls weekly, while a mere 5% reported fewer than six.

It is pertinent to note that the participants predominantly qualified as first-time users of VR technology. Those with prior VR experience reported limited exposure and unfamiliarity with the Horizon Workrooms software.

All methods were approved by the University of Trento, Human Research Ethics Committee (Protocol 2022-037). The whole procedure was realized in accordance with the Helsinki Declaration. Informed consent was obtained by all participants prior to the experiment. No financial incentives were offered for participation.

### Experimental design

The study employed a within-subjects design framework, wherein each participating group was exposed to three distinct interaction modalities: VC, FTF, and immersive experiences in VR environment. To account for potential order effects, the sequence of these conditions was randomized across groups. However, logistical constraints necessitated that the VR and FTF sessions be conducted consecutively on the same day, thereby precluding the possibility of interspersing the VC condition between them.

For the purpose of interaction analysis, comprehensive video recordings of all sessions were captured. This was facilitated through the utilization of Free Cam 8, a specialized software designed for screen recording, capable of capturing both visual and auditory data.

VC sessions were conducted remotely via Microsoft Teams. Participants had the latitude to join these sessions from locations of their preference, utilizing their personal computing devices. Conversely, FTF and VR sessions were predominantly orchestrated within the premises of Terna S.p.A.

During FTF interactions, the existing technological infrastructure within the meeting rooms was leveraged. This included a large display screen, a webcam, and an array of microphones, which not only facilitated task explanation by the experimenter but also enabled video capture via screen recording software. Participants were seated in a horseshoe-shaped arrangement around a table, within a room specifically designated for meetings by the organization.

For the VR sessions, Oculus Quest 2 headsets were employed, selected for their market prevalence and functional versatility. The VR meeting platform of choice was Horizon Workrooms, a software designed for corporate virtual interactions. Within this platform, a "mountain cabin" setting was selected for the study. Avatars, meticulously designed to resemble participants based on photographic references, were positioned in a manner analogous to their physical seating arrangement to minimize audio reverberation.

Prior to the commencement of each VR session, a 30-min orientation was conducted by a designated company representative. This orientation was bifurcated into two segments: the first half elucidated the hardware functionalities in a real-world setting, while the latter half was conducted within the VR environment to familiarize participants with the software interface. This was particularly crucial for the effective utilization of Horizon Workrooms' interactive tools, such as the shared whiteboard. Although the primary responsibility for idea documentation was allocated to a single participant, all group members were trained in the tool's usage to mitigate potential technical disruptions, given the beta status of the software.

Three discussion topics were selected for the experimental tasks, each designed to be sufficiently generic to facilitate broad engagement and ideation. These topics were presented with a specific question to be addressed by the participants:

- **Tourism**: “What could be done to improve and/or increase tourism in the Italian state?"
- **Restaurant**: “A restaurant in the city center is experiencing a drop in customers, what can they do to increase them?”
- **Pollution**: “Pollution is a global problem, what could be done to improve the situation?”

The sequence of these topics was also randomized to control for potential order effects, thereby ensuring a balanced distribution of interaction modalities across topics.

### Procedure

Each experimental session adhered to a uniform structure, lasting approximately one hour and encompassing the following sequential phases:

- **Orientation and topic assignment**: Participants were initially briefed on the specific activity they would engage in following a standard illustrative hands-on procedure, along with the thematic focus designated for that particular session.
- **Idea generation**: A 20-min interval was allocated for the uninhibited generation of ideas pertaining to the assigned topic. Within each group, a designated individual was responsible for documenting the emergent ideas. The documentation medium varied depending on the interaction modality: traditional pen-and-paper for FTF sessions, a communal chat feature within several platforms for VC, and a virtual blackboard manipulated via handheld controllers in the VR environment. Importantly, participants were explicitly instructed to abstain from evaluative judgments during this ideation phase, focusing solely on the capture of emergent ideas.
- **Idea prioritization**: Subsequent to the ideation phase, participants collaboratively engaged in the formulation of a ranked list featuring the top five ideas. This ranking process was to be consensually agreed upon within a maximum time frame of 20 min. Upon completion, the group communicated their finalized list to the experimenter, who then terminated the timing mechanism and recorded the elapsed duration.
- **Questionnaire administration**: Participants proceeded to complete a series of questionnaires, accessed via a hyperlink disseminated through email by the researcher. The estimated time for questionnaire completion did not exceed 10 min.

### Measures

Below we list all the dimensions evaluated in this research with the related investigation tools used. All the questionnaires used were translated into Italian and completed at the end of each session by the participants.

### Flow short scale (FSS)

The Flow Short Scale^38^ is a self-assessment test that investigates the state of flow. It consists of 13 items, to be answered as much as you agree with a 7-point Likert scale, with the labels corresponding to 1 “Not at all”, 4 “Partially” and 7 “Very much”. The 4 subscales of the test measure the variables of: flow experience, perceived outcome importance, fluency of performance, absorption by activity.

### Zoom exhaustion and fatigue scale (ZEF Scale)

The Zoom Exhaustion and Fatigue Scale^36^ is a self-assessment test used to evaluate the perception of fatigue during the use of digital communication tools, in particular in a videocall. The test consists of 15 items, which investigate 5 dimensions of fatigue: general, social, emotional, visual, and motivational fatigue. The respondent indicates how much he feels the sensation indicated on a 5-point Likert scale, indicated with the labels: "Not at all", "Slightly", "Moderately", "Very" and "Extremely".

The questionnaire was adapted for each type of session. For the FTF session it was decided to exclude the 3 items investigating visual fatigue, as no device with a screen was used for the FTF activity. Then the visual fatigue variable was only compared between the VR and VC sessions.

### Perceived performance scale (PPS)

The Perceived Performance Scale^39^ is a self-assessment test, it is used to assess the participants' perception of their performance during the activity. The questionnaire consists of 5 items. Participants rate how much they agree with the statements on a 7-point Likert scale. In the extremes are the labels “Strongly disagree” and “Strongly agree”. The first three items specifically investigate how much the participants perceive they were productive during the activity. The last two items instead investigate the weight that the participants attribute to the other group members on their group performance, asking them if they think they would have generated more ideas or if these would have been of higher quality with different group members.

### Single-item social identification (SISI)

The Single-Item Social Identification^40^ is a self-assessment test. The item reports “I felt part of the group”, to which the participants respond by indicating the value on a 7-point Likert scale, with 1 “Not at all” and 7 “Completely” indicated at the extremes.

### Interaction process analysis (IPA)

To investigate the dynamics of group interaction, the Bales Interaction Process Analysis evaluation grid was used^35^. The researcher observed the video recordings of all the sessions and whenever any form of interaction occurred between the members of the group this was reported in one of the 12 categories that make up the grid. In this way we obtain the frequency of implementation of that behavior. The categories are then grouped into 4 survey areas: “Positive Socio-Emotional Area”, “Negative Socio-Emotional Area”, “Task Orientation Area: response attempts”, and “Task Orientation Area: questions”.

### Creativity

The creativity component was evaluated by comparing the average number of ideas generated for each type of session. A second analysis was then done using the OSF tool for the assessment of divergent thinking. Two researchers assigned a score to each idea generated for the three variables:

- *Uncommon*: indicates how unique the idea is compared to the other ideas generated by the sample. For example, a score of 5 indicates that no one else has reported the same thought
- *Remote*: refers to how much that idea deviates from common thought in general, how remote it is
- *Clever*: the qualitative level of the idea is evaluated, in terms of complexity, formulation and intelligence

### Decision making

Decision making within the groups was evaluated calculating the ratio between the time required to draw up the ranking of the 5 best ideas and the number of ideas produced in that session. This allowed us to obtain the average time required by the group to evaluate each idea that emerged, then for the comparison we considered the means of all the sessions for each condition.

### Data analysis

All statistical computations were executed utilizing R software (version 4.2.3) and Microsoft Excel.

Prior to conducting comparative analyses across experimental conditions, an initial assessment of data normality was performed. Specifically, the Shapiro–Wilk Normality Test was applied to each data subset to ascertain the distributional characteristics and identify any non-parametric distributions. This preliminary step informed the selection of appropriate statistical tests for subsequent analyses.

For data subsets conforming to parametric distributional assumptions, a repeated-measures Analysis of Variance (ANOVA) was employed. This was justified by the repeated-measures design, wherein each participant—and by extension, each group—engaged in identical activities across the three conditions (VC, VR, and FTF). A *p*-value threshold of less than 0.05 was considered indicative of statistical significance, thereby warranting the rejection of the null hypothesis positing equivalence across conditions. To control for Type I error arising from multiple comparisons, False Discovery Rate (FDR) correction was applied to all resulting p-values. Post-hoc pairwise comparisons were conducted using *t*-tests for dependent samples, focusing on the following condition pairs: VC-VR, VR-FTF, and VC-FTF.

Conversely, for data subsets exhibiting non-parametric distributional characteristics, the Friedman test was utilized as a non-parametric alternative to ANOVA for repeated measures. Subsequent pairwise comparisons were conducted using the Wilcoxon Signed-Rank Test for paired samples, with the resulting p-values serving as indicators of statistical significance. Test for paired samples, and these p-values were likewise adjusted using FDR correction to ascertain statistical significance.

Additionally, Pearson correlational analyses were conducted to explore the relationship between participants' age and their questionnaire responses across different session types. Regression analyses were performed using Microsoft Excel, with *p*-values less than 0.05 deemed statistically significant.

## Data Availability

The datasets generated and analysed during the current study are not publicly available since participants did not provide explicit written consent regarding the sharing of their data on public repositories, but are available from the corresponding author on reasonable request.
